# Autoregulation monitoring and outcome prediction in neurocritical care patients: Does one index fit all?

**DOI:** 10.1007/s10877-015-9726-3

**Published:** 2015-06-18

**Authors:** Bernhard Schmidt, Matthias Reinhard, Vesna Lezaic, Damian D. McLeod, Marco Weinhold, Heinz Mattes, Jürgen Klingelhöfer

**Affiliations:** Department of Neurology, Chemnitz Medical Centre, Dresdner Str. 178, 09131 Chemnitz, Germany; Department of Neurology, University of Freiburg, Breisacher Str. 64, 79106 Freiburg, Germany; School of Biomedical Sciences and Pharmacy, The University of Newcastle, Callaghan, NSW 2308 Australia; Department of Electrical, Electronic and Communication Engineering, Friedrich-Alexander Universität Erlangen-Nürnberg, 91052 Erlangen, Germany

**Keywords:** Cerebral autoregulation, Cerebrovascular pressure reactivity, Modified Rankin Scale, Cerebral blood flow, Traumatic brain injury, Stroke

## Abstract

Indexes PRx and Mx have been formerly introduced to assess cerebral autoregulation and have been shown to be associated with 3-month clinical outcome. In a mixed cohort of neurocritical care patients, we retrospectively investigated the impact of selected clinical characteristics on this association. Forty-one patients (18–77 years) with severe traumatic (TBI, N = 20) and non-traumatic (N = 21) brain injuries were studied. Cerebral blood flow velocity, arterial blood pressure and intracranial pressure were repeatedly recorded during 1-h periods. Calculated PRx and Mx were correlated with 3-month clinical outcome score of modified Rankin Scale (mRS) in different subgroups with specific clinical characteristics. Both PRx and Mx correlated significantly with outcome (PRx: r = 0.38, *p* < 0.05; AUC = 0.64, n.s./Mx: r = 0.48, *p* < 0.005; AUC = 0.80, *p* < 0.005) in the overall group, and in patients with hemicraniectomy (N = 17; PRx: r = 0.73, *p* < 0.001; AUC = 0.89, *p* < 0.01/Mx: r = 0.69, *p* < 0.005; AUC = 0.87, *p* < 0.05). Mx, not PRx, correlated significantly with mRS in patients with heart failure (N = 17; r = 0.69, *p* < 0.005; AUC = 0.92, *p* < 0.005), and in non-traumatic patients (r = 0.49, *p* < 0.05; AUC = 0.79, *p* < 0.05). PRx, not Mx, correlated significantly with mRS in TBI patients (r = 0.63, *p* < 0.01; AUC = 0.89, *p* < 0.01). Both indexes did not correlate with mRS in diabetes patients (N = 15), PRx failed in hypocapnic patients (N = 26). Both PRx and Mx were significantly associated with 3-month clinical outcome, even in patients with hemicraniectomy. PRx was more appropriate for TBI patients, while Mx was better suited for non-traumatic patients and patients with heart failure. Prognostic values of indexes were affected by diabetes (both Mx and PRx) and hypocapnia (PRx only).

## Introduction

**Fig. 1:**
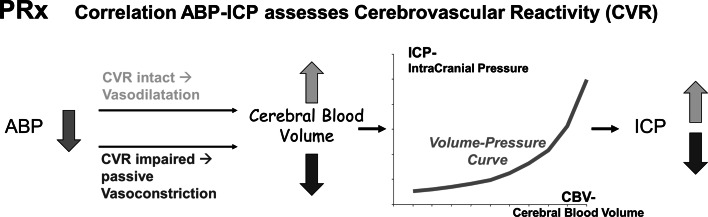
Physiologic model conception of PRx. If cerebrovascular pressure reactivity (CVR) is intact (*upper line*), small cerebral vessels dilate in response to decreasing ABP, resulting in an increased cerebral blood volume. In regards to the pressure–volume curve of brain [22–24], this causes an increase of ICP, i.e. ABP and ICP are negatively correlated. If CVR is disturbed (*lower line*), ABP decrease is passively followed by constriction of small vessels. This causes a decrease of cerebral blood volume, and, therefore, causes a decrease of ICP. ABP and ICP are positively correlated. Conversely, in the case of increasing ABP, a negative correlation between ABP and ICP is generally associated with intact CVR, while a positive correlation between both signals indicates impaired CVR

The purpose of cerebral autoregulation (CA) is to keep cerebral blood flow (CBF) constant during variations of cerebral perfusion pressure (CPP). A pressure reactivity index PRx and a CBF related index Mx have been formerly introduced to assess CA [1–5] in patients with acute severe cerebral diseases. Although the pathophysiologic basis of both indexes is not completely clear, the index PRx [5] is assumed to describe the response of small cerebral vessels to spontaneous changes of arterial blood pressure (ABP) in terms of changes of intracranial pressure (ICP), the so-called cerebrovascular pressure reactivity (CVR) (Fig. 1). The index Mx [4] describes the effect of spontaneous changes in CPP (=ABP–ICP) on the transcranial Doppler assessed cerebral blood flow velocity (CBFV) in the middle cerebral artery (MCA). Assuming the vessel diameter of MCA to be almost constant in time [6], changes of CBFV may be seen as a surrogate for changes of CBF. Therefore, synchronous changes of CPP and CBFV may indicate a lack of CA.

In former studies with traumatic brain injury (TBI) patients, PRx coincided with classic measures of CA such as the lower limit of autoregulation [7], and both PRx and Mx were associated with clinical outcome [8–11]. In patients with intracerebral hemorrhages a significant association between unfavourable outcome and increased PRx [12] as well as increased Mx [13] was shown. However, in a study with aneurysmal subarachnoidal hemorrhage (SAH) patients, clinical outcome and PRx did not correlate [14]. Recently, PRx has been used in the individual management of CPP in neurocritical care patients (so-called ‘optimal CPP’) [15–18].

Until now, little is known about the influence of primary diseases, co-morbidities and other risk factors such as age and neurosurgical interventions on the outcome predictive value of autoregulatory indexes. PRx and Mx describe different aspects of autoregulation, and therefore, are likely to be differentially influenced by such clinical characteristics. The primary aim of the present study was to investigate the impact of selected clinical characteristics on the association of both autoregulation indexes Mx and PRx with 3-month clinical outcome. The secondary aim was to confirm the formerly reported association between autoregulation indexes and clinical outcome [7–12] and investigate whether this association was still valid in a cohort of neurocritical care patients with very different types of brain injury (TBI, haemorrhagic stroke, and others). Both indexes were analysed in view of their suitability for outcome prediction in different subgroups of patients with specific clinical characteristics.

## Materials and methods

### Patient population

In a retrospective study, recorded signal data from 41 consecutive patients with severe cerebral diseases (age 18–77 years, mean 52 ± 17 years, 28 male/13 female) who underwent multimodal monitoring between 2005 and 2009 were analyzed. Part of the study population had been included in previous analyses focusing on different aspects on CA monitoring [19]. Patients were treated in the Neurocritical Care Unit of the Chemnitz Medical Centre. They suffered either from TBI (N = 20) with subarachnoidal hemorrhages (N = 7), intracerebral hemorrhages (N = 4) and intracranial hematoma (N = 11), or from non-traumatic diseases (N = 21), i.e. aneurysmatic subarachnoidal hemorrhages (N = 4), spontaneous intracerebral hemorrhages (N = 10), MCA infarction (N = 4), cerebral venous sinus thrombosis, hypoxic encephalopathy, and encephalitis. In 19 patients, hemicraniectomy was performed. During time of data recording all patients were sedated and mechanically ventilated with ventilator settings fixed during recording time. Patients’ arterial partial pressure of CO_2_ (PaCO_2_) ranged from 26 to 49 mmHg. Patient management procedures included the maintenance of CPP above 60 mmHg.

The study was approved by the Local Ethics Committee. All signal monitoring was part of a clinical routine. The retrospective data analysis did not require individual consents.

### Monitoring

A 2 MHz pulsed Doppler device (Multidop-P, DWL, Sipplingen, Germany) was used for assessment of transcranial Doppler (TCD) signal. The envelope curve of CBFV in the middle cerebral artery (MCA) was continuously monitored in the hemisphere ipsilateral to the brain lesion in most cases. TCD signals were recorded during stable periods free from nursing. ABP was measured with a standard manometer line inserted into the radial artery. ICP was measured using either implanted intraparenchymal or intraventricular microsensor catheters (Raumedic GmbH, Helmbrechts, Germany). ICP assessed by external ventricular drain was not considered for recording.

### Computer-assisted recording

Personal computers fitted with data acquisition systems (Daq112B, Iotech, Inc., Cleveland, OH, USA) and software developed in-house [20] were used for recording and analyzing TCD, ABP and ICP signals and for calculation of PRx and Mx (see below). For each recording time point, signals were assessed over a 60 min period with a sampling frequency of 25 Hz. If possible, recording was repeated at days 2, 4, and 7. In total 130 recordings of 41 patients were acquired.

### Calculation of indexes PRx and Mx

PRx and Mx were calculated retrospectively. Initially, the recorded signal data of CBFV, ICP and ABP was averaged over 10-s intervals in order to erase oscillations from mechanical ventilation and higher frequencies. Pearson’s correlation coefficients were calculated from thirty consecutive average signals of ABP and ICP. The step length was 10 s, i.e. correlations were performed over time periods of 5 min. This calculation was repeated every minute. The computed correlation indices were averaged and resulted in the pressure reactivity index PRx [5].

Mx was calculated completely analogous to PRx by correlating averaged CBFV and CPP values instead of ABP and ICP values [4].

Zero or negative values of these indexes indicate active regulation of blood flow (Fig. 1) [4, 5], while positive index values suggest impairment of flow regulation.

**Fig. 2:**
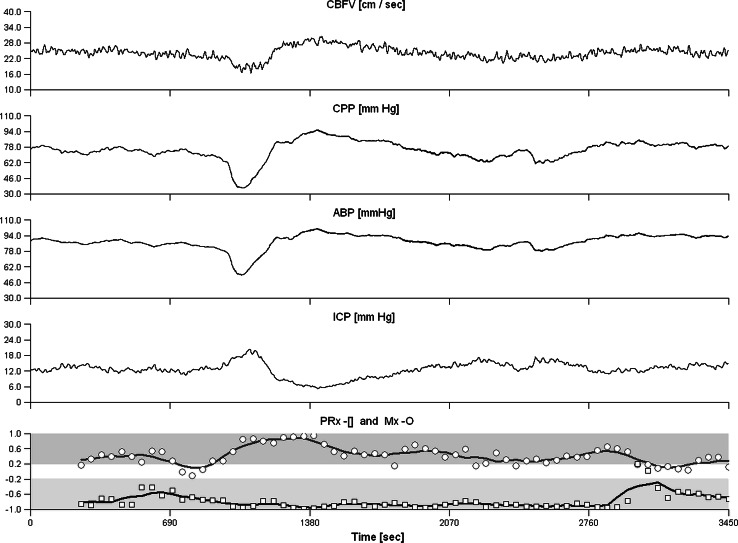
Signal recording of a 71-year-old patient with hemorrhagic stroke, heart failure, and a 3-month outcome mRS score of 4. CBFV, ABP and ICP have been recorded for 3450 s. CPP was calculated by ABP–ICP. In the lower channel, signal correlation coefficients are indicated either by *circles* (between CBFV and CPP, for Mx calculation) or by *squares* (between ABP and ICP, for PRx calculation) and moving average curves of five consecutive correlation coefficients are drawn. The signals CBFV and CPP showed strictly parallel fluctuations, while signal changes of ABP and ICP were clearly opposed. Accordingly, the indexes strongly differed: Mx was 0.31, indicating impaired CA, while PRx was −0.77, indicating intact cerebrovascular reactivity. The moderately severe outcome (mRS score = 4) better fits to the Mx value. *ABP* arterial blood pressure, *CA* cerebral autoregulation, *CPP* cerebral perfusion pressure, *CBFV* cerebral blood flow velocity, *ICP* intracranial pressure, *mRS* modified Rankin Scale

One PRx and one Mx value were calculated for each signal recording (Fig. 2). For outcome analysis, these index values were averaged over all recordings of each patient thus resulting in one PRx and one Mx per patient.

### Association with clinical outcome and statistics

The association of CA-indexes with 3-month clinical outcome on the whole population including non-survivors was assessed by:

1. The Pearson correlation after Mudholkar test [21] for bivariate normal distribution between each index and the mRS values, and
2. The area under the curve (AUC) of a ROC curve analysis between index values and poor outcome (score of mRS ≥ 4). The significance of deviation of ROC curve from 50 % line was assessed by Wilcoxon–Mann–Whitney test. AUC connected with Mx and PRx were compared using a method described by DeLong et al. [22].

The association of CA-indexes with in-hospital mortality was measured in terms of

1. A logistic regression between the CA-indexes and mortality, and
2. The AUC of a ROC curve analysis between index values and in-hospital mortality.

In both cases, the index was denoted as having predictive value (of either poor 3-month outcome or in-hospital mortality), if the corresponding statistical tests were significant and AUC was above 0.7.

The weaker term “associated with outcome” denoted significance of any of the performed statistical tests of relationship between index and outcome.

In basic clinical data (Table 1), categorical comparison was provided by Fisher’s exact test. Numerical characteristics were compared by unpaired *t* test after Kolmogoroff–Smirnoff test of normal distribution, association with outcome was assessed by logistic regression analysis. Significance of shift of mRS scores in patients with low indexes compared to those with high index values was assessed by Mantel–Haenszel test.

**Table 1:** Basic clinical data and association with in-hospital mortality and 3-month outcome

Characteristics	All patients N = 41	In-hospital mortality		Three-month clinical outcome (mRS)	
		Survival; death N = 35; 6	Logistic regression OR; 95 % CI	Good; poor outcome N = 14; 22	Logistic regression OR; 95 % CI
Age	52 ± 16	51 ± 16; 60 ± 10	1.04; 0.98–1.11	46.9 ± 16.0; 56.6 ± 15.6	1.04; 0.99–1.09
Age (with PRx)					1.03; 0.98–1.08
Age (with Mx)					1.02; 0.97–1.08
Female	13	12; 1	2.6; 0.26–26.40	4; 9	0.7; 0.16–3.1
Hemicraniectomy	19	18; 1	5.3; 0.53–53.0	7; 12	1.2; 0.30–4.78
TBI	20	16; 4	1.45; 0.42–4.95	6; 14	1.32; 0.97–1.80
Heart failure	18	17; 1	4.72; 0.47–47.23	6; 12	0.75; 0.19–3.00
Diabetes mellitus II	15	12; 3	0.52; 0.09–3.12	5; 10	0.67; 0.16–3.00
Alcohol abuse	9	4; 5***	38.8; 3.36–447***	2; 7	2.25; 0.37–13.8
PaCO_2_ (mmHg)	37.0 ± 4.7	37.1 ± 5.0; 36.7 ± 2.1	0.98; 0.80–1.21	37.2 ± 6.4; 37.5 ± 3.7	1.02; 0.88–1.18
ABP (mmHg)	89 ± 11.2	90 ± 11.3; 85 ± 9.7	0.96; 0.89–1.05	91.0 ± 12.7; 86.0 ± 10.7	0.96; 0.90–1.02
ICP (mmHg)	12 ± 9.3	11 ± 3.8; 20 ± 20.6	1.11; 0.96–1.28	10.7 ± 4.3; 13.1 ± 12.4	1.03; 0.93–1.14
PRx	0.12 ± 0.27	0.07 ± 0.22; 0.41 ± 0.33***	1.71; 1.09–2.69*	0.04 ± 0.24; 0.22 ± 0.28	1.32; 0.97–1.80
PRx (with age)					1.26; 0.92–1.72
Mx	0.09 ± 0.26	0.06 ± 0.23; 0.28 ± 0.40	1.40; 0.89–2.18	−0.07 ± 0.21; 0.21 ± 0.27***	1.64; 1.13–2.37**
Mx (with age)					1.57; 1.07–2.28*

Clinical data refer to the complete patient group (col. 2), as well as to the subgroups of patients specified by in-hospital mortality (col. 3) and 3-month outcome (col. 5). Data is in terms of occurrence or mean value ± SD. Impact on outcome was assessed by Fisher’s exact test, unpaired *t* test (cols. 3, 5), or univariate logistic regression analysis (cols. 4, 6). In addition, impact of both Mx and PRx on 3-month outcome was assessed age-corrected by bivariate (PRx and Age, as well as Mx and Age) logistic regression. Alcohol abuse and high PRx, but not Mx, were significant risk factors for mortality, while Mx, but not PRx, was a significant predictor of poor outcome. High PRx and alcohol abuse were not associated (*p* > 0.2, Fisher’s exact test)

*OR* odds ratio, *mRS* modified outcome scale, mRS ≥ 4 denotes poor outcome

Significance levels: * *p* < 0.05; ** *p* < 0.01; *** *p* < 0.005; *** *p* < 0.001

## Results

Baseline clinical characteristics of the studied patients are shown in Table 1.

*In*-*hospital mortality* Six of the patients died in-hospital. Reasons for death were increased ICP causing cessation of cerebral blood flow (N = 2), decompensated hepatic insufficiency (N = 2), and pulmonary embolism (N = 2). On average, both PRx and Mx indexes were higher in the Non-Survivors group than in the Survivors group, the difference being significant only in the case of PRx (Table 1). Univariate logistic regression (ULR) analysis found alcohol abuse and PRx, but not Mx, as significant risk factors for mortality (Table 1). ROC curve analyses between PRx and mortality yielded 0.2 as the critical threshold (CT) between low and high PRx; the AUC was 0.79. In addition, the day-1 assessed PRx (PRx_day1) as well as the maximum of the two PRx values calculated on day 1 and on day 2 (PRx_max1&2) were tested for association with mortality. Similar to (the averaged) PRx, both PRx_day1 and PRx_max1&2 showed significant association with in-hospital mortality (ULR: *p* < 0.05; PRx_day1: AUC = 0.85; PRx_max1&2: AUC = 0.74). In case of Mx, AUC was 0.70, and 0.38 was the CT. Using both CT, the associations between high PRx and mortality as well as high Mx and mortality were significant (*p* < 0.05, Fisher’s exact test).

**Fig. 3:**
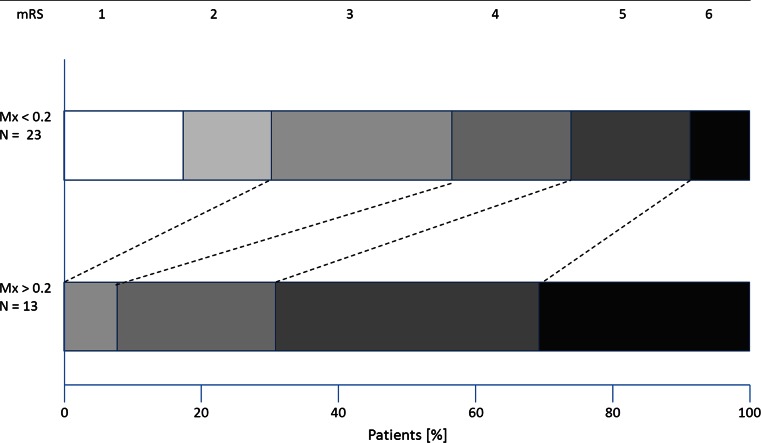
Three-month outcome in patients with high Mx and with low Mx. In patients with low Mx (Mx < 0.2; N = 23) the distribution of mRS scores (*upper bar*) was shifted towards lower scores (indicating better outcome) if compared to the mRS scores of the patients with high Mx (Mx ≥ 0.2; N = 13; *lower bar*). In seven patients mRS was either 2 or 1, in all of them Mx was low. The difference between outcome distributions of both groups was significant (*p* < 0.005; Mantel–Haenszel test). *mRS* modified Rankin Scale, *0* no symptoms–*6* death)

**Fig. 4:**
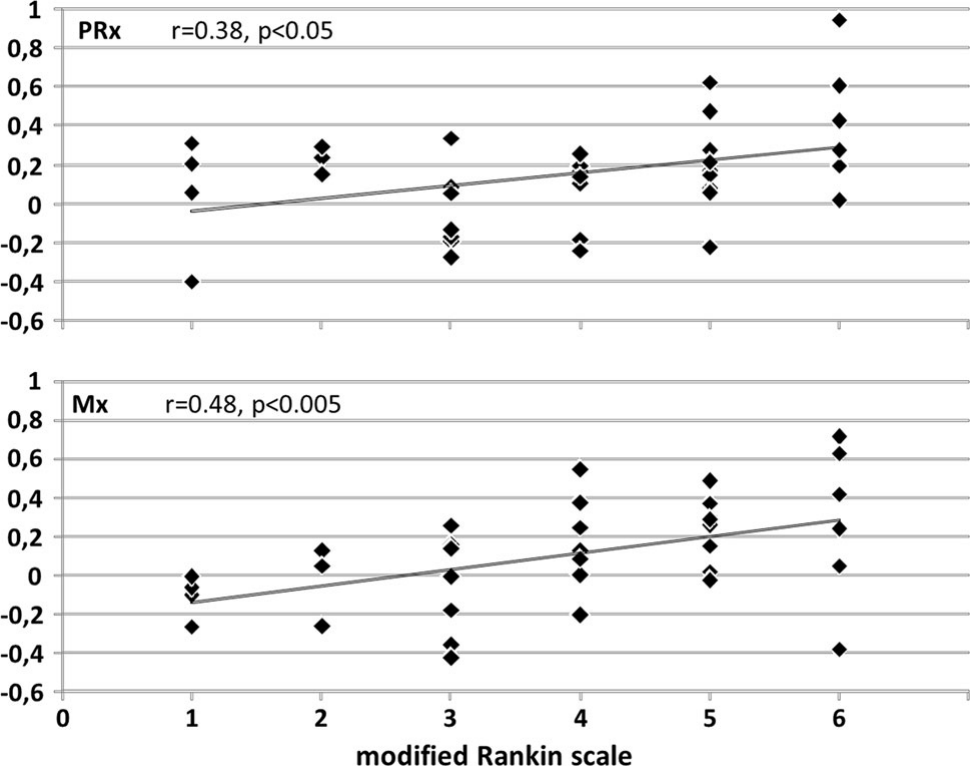
PRx and Mx *plotted* versus modified Rankin Scale (mRS). In the subgroup of 36 patients with known 3-month outcome, higher index values corresponded to poorer outcome. mRS scores were significantly correlated with PRx (r = 0.38, *p* < 0.05), and even stronger correlated with Mx (r = 0.48, *p*< 0.05)

*Three*-*month clinical outcome* In 36 patients, the 3-month clinical outcome in terms of scores of modified Rankin Scale (mRS) could be assessed. ULR analysis found Mx, but not PRx, as the only significant risk factors for bad outcome (Table 1, col. 6). Significance of Mx remained even if corrected to age (Table 1). A ROC curve analysis yielded 0.2 as the CT of Mx for prediction of bad outcome (mRS 4–6); AUC was 0.80. For PRx, the CT was 0.1 with AUC 0.64. High Mx (>0.2) was associated with unfavourable outcome (*p* < 0.005; Fisher’s exact test), while high PRx (>0.1) was not. The patients with low Mx showed a significant shift towards favourable outcome scores (*p* < 0.005; Mantel–Haenszel test) (Fig. 3), in contrast to low PRx where this shift effect was not significant. Both PRx and Mx correlated significantly with mRS score (PRx: r = 0.38, *p* < 0.05; Mx: r = 0.48, *p* < 0.005) (Fig. 4; Table 2). *Predictive value of autoregulation indexes in clinical subgroups.* PRx correlated significantly with mRS in TBI patients but not in patients with non-traumatic diseases (Table 2). Mx behaved conversely. Both PRx and Mx correlated with mRS in patients with hemicraniectomy (N = 19). Neither PRx nor Mx correlated with mRS in patients without hemicraniectomy, in older patients (>60 years; N = 14) and in patients with diabetes mellitus (N = 15). In patients with congestive heart failure (NYHA state I or higher; N = 18), Mx but not PRx correlated with mRS (Fig. 2). AUC of Mx ranged from 0.71 (in patients below 60 years) to 0.92, AUC of PRx ranged from 0.64 (in total population) to 0.89 (Table 2). Mx and PRx did not significantly correlate in patients above 60 years and in heart failure patients. Compared to Mx and PPx, non-averaged index values such as e.g. PRx_day1 showed clearly lower association to 3-month outcome and were not presented here. Detailed results are provided in Table 2.

**Table 2:** Association of Mx and PRx with 3-month outcome in various patient subgroups

Subgroup specification	Correlations			
	Number of patients	Pearson correlation coefficient; AUC of ROC curve; critical threshold		
	N; N(mRS)	mRS–Mx	mRS–PRx	Mx–PRx
	41; 36	0.48***; 0.80***; 0.20	0.38*; 0.64; 0.10	0.55***
Type of disease				
TBI	20; 16	0.49	0.63**; 0.89**; −0.06	0.56**
Non-TBI	21; 20	0.49*; 0.79*,#; 0.14	0.09	0.55**
Age				
>60 years	14; 13	0.18	0.44	0.06
<60 years	27; 23	0.43*; 0.71	0.30	0.62***
PaCO_2_ (mmHg)				
26–40	31; 26	0.55***; 0.77*; 0.14, 0.59	0.31	0.57***
30–49	39; 34	0.59***; 0.82***; 0.20	0.47**; 0.69	0.58***
35–49	27; 24	0.68***; 0.92***; 0.00	0.57***; 0.76	0.68***
Heart failure				
Yes	18; 17	0.69***; 0.92***; 0.00	0.34	0.37
No	23; 19	0.38	0.43	0.63***
Hemicraniectomy				
Yes	19; 17	0.69***; 0.87*; 0.26	0.73***; 0.89**; 0.07	0.58**
No	22; 19	0.41	0.10	0.53**
Diabetes mellitus II				
Yes	15; 15	0.20	0.41	0.57*
No	26; 21	0.68***; 0.87***; 0.00	0.36	0.57***

In the complete patient population and in various subgroups correlations were calculated between mRS and Mx and PRx as well as between PRx and Mx. If the index correlated with mRS and number of cases was at least 15, AUC with critical threshold, sensitivity and specificity of index for prediction of poor outcome (mRS ≥ 4) were presented. PRx and Mx mutually correlated except in patients above 60 years and in patients with heart failure. Mx did not correlate with mRS in patients above 60, in patients with diabetes, and in patients without heart failure. PRx correlated with mRS in TBI patients. PRx did not correlate with mRS in non-traumatic patients, in age-specified subgroups, and in the heart failure and diabetes related subgroups. In patients with hemicraniectomy, both PRx and Mx correlated with mRS, but did not in patients without hemicraniectomy. Predictive value of indexes was higher in normal PaCO_2_ range (>35 mmHg) than in patients with low PaCO_2_ (26–35 mmHg). Although AUC clearly differed between Mx and PRx, the difference was significant only in the group of non-traumatic patients

*AUC* area under the curve; correlation: for simplicity denotes a significant correlation, *mRS* modified Rankin Scale, *N* population size; *N*(*mRS*) number of patients with known 3-month mRS score

Significance levels: * *p* < 0.05; ** *p* < 0.01; *** *p* < 0.005; *** *p* < 0.001; ^#^ significance of Mx–PRx difference in AUC (*p* < 0.05)

## Discussion

In this study, CA related indexes were applied to patients with both traumatic and non-traumatic diseases. Despite this heterogeneity of diseases, both PRx and Mx were significantly associated with clinical outcome. CA is a central mechanism in brain and its failure might be a pathology in itself within any causing disease. However, some of our results suggested an impact of the underlying diseases. In particular, PRx fitted better to TBI patients, while Mx had a stronger correlation with outcome in patients with non-traumatic diseases.

### PRx and Mx focus on different aspects of autoregulation

Hyperventilation, heart failure, and hemicraniectomy clearly influenced the predictive value of PRx and Mx. In hyperventilated patients, the cerebral vasoconstriction effect of low PaCO_2_ may have impaired the predictive value of PRx. In heart failure, insufficient cardiac output and autoregulatory failure was detected by Mx, which assesses blood flow dynamics, but was not detected by PRx. This may explain why PRx and Mx did not correlate in patients with heart failure and why only Mx was predictive in these patients. The observation of improvement in PRx in hemicraniectomy patients confirmed [23] and contradicted [24] former reports. The strength of ICP reactions to ABP changes depends on the current slope of the intracranial pressure–volume curve (Fig. 1). Therefore, PRx is influenced by cerebral compliance (CC), which is the reciprocal of this slope [26, 27]. While CC may vary in patients without hemicraniectomy, it may be assumed that this co-factor of PRx remains similar in all patients with hemicraniectomy. This might explain why PRx was an improved predictor of mRS in patients with hemicraniectomy and failed in patients without. However, only six of the 19 patients without hemicraniectomy suffered from TBI, which suggests a contribution of non-traumatic diseases to the failure of PRx in this group. Therefore, these results do not contradict a previously reported significant association of PRx with outcome in purely TBI patient groups without hemicraniectomy [8, 11]. The differences of predictive values between patients with and without hemicraniectomy were less pronounced in Mx. The confounding effect of CC on PRx was previously used to explain discrepancies between Mx and PRx during increase of ICP [28].

### Role of early versus late outcome parameters

Mx was a better predictor of 3-month mRS than PRx, while PRx was superior to Mx in predicting the early in-hospital mortality. The reason for this imbalance is unclear. Similar observations were made in recent studies with PRx and Mx in TBI patients only [11, 28]. It might be that to some extent disturbance of CVR (assessed by PRx) is a priori associated with affected brain viability, while impaired CBF (assessed by Mx) may cause secondary damage, which affects outcome but predominantly may not be lethal. Following this hypothesis, Mx-controlled management of CPP would appear particularly promising. However, due to the problems with long-term TCD insonation, clinical interventions have been focusing so far on the setting of PRx-optimized CPP [15–18].

### Limitations

Our study included a small number of patients. Confirmation of the results using larger populations is necessary. For sub-group analyses, ‘clinical characteristics’ were defined as cardiovascular risk factors that had the potential to affect patient outcome. However, adequate incidence of each risk factor was essential for statistical analysis. For this reason, not all cardiovascular risk factors could be analysed e.g. ‘alcohol abuse’ was analysed as there were nine reported events; ‘smoking’ could not be analysed because it was not consistently reported. In our hospital, we tested CA but did not continuously monitor it. Therefore, additional information such as the time duration of increasing indexes was not available. Recently this parameter was shown to correlate with 3-month outcome [16]. In our study we used averaged values of Mx and PRx and correlated these values with outcome. A general drawback of this method is that it neglects time-related changes in autoregulation and in some cases may lead to the ‘averaging out’ of occasional extreme index values. This is clearly a weakness of this kind of approach. On the other hand, averaged PRx or Mx, in previous studies [9–12] were found to be independent predictors of outcome, therefore, in the present study we relied on this method. Moreover, association of these averaged indexes with outcome was confirmed by our own results. In cases of in-hospital mortality we also studied non-averaged PRx values, because averaging the index over several days for prediction of a possible sudden event does not seem to be clinically useful. However, we found an association of these parameters with mortality as well.

In our study, independence of autoregulation indexes from other clinical parameters could not be stated due to a limited number of events, thus limiting the application of multivariate logistic regression [29]. In our study, 14 patients had a favourable outcome. Therefore, even if *relaxing* the commonly used rule of ten events per independent variable [29], we could not consider more than two risk factors for logistic regression with the target variable ‘good/bad outcome’. However, we investigated patients’ age as joined risk factor. Another *natural* risk factor, the Glasgow Coma Score on admission was not sufficiently documented for evaluation.

Deviation of CPP from CPPopt was formerly reported to be associated with bad outcome [15, 16]. However, we could not include this parameter in our analysis. Our recordings at each time point were limited to 1-h periods. This duration was too short for calculating CPPopt. Although being visibly different, comparison of AUC for 3-month outcome showed a significant difference between PRx and Mx only in the group non-traumatic patients. This might be caused by the small sizes of investigated subgroups. We had access to a detailed mRS score in only 36 patients. This produced a predominance of in-hospital fatal outcomes; all fatalities were reported. In contrast to other studies [11, 25], ICP was not a risk factor of poor outcome. However, ICP was elevated (>20 mmHg) in only one of our patients.

The primary cerebral disease state directly caused death in only two of the study patients. The question may arise whether there is any logic in considering brain-derived indexes such as PRx and Mx in patients who died from multi-organ failure after hepatic insufficiency or pulmonary embolism. However, reasons for death are complex and multi-factorial. In our study, we found a significant association between CA-indexes and death, but we could not provide evidence of causality.

## Summary

Both PRx and Mx were significantly associated with in-hospital mortality and 3-month clinical outcome. PRx was more strongly associated with in-hospital mortality than Mx, while Mx was superior in prediction of functional outcome after 3 months. PRx was a predictor of 3-month outcome in TBI patients, but was not suitable for non-traumatic patients or patients with heart failure. Mx was a predictor of 3-month outcome in non-traumatic patients and in patients with heart failure. Both indexes were suitable for patients with hemicraniectomy. In patients older than 60 years and in patients with diabetes, neither PRx nor Mx was associated with outcome. Predictive value PRx was best if PaCO_2_ was kept above 35 mmHg. If PRx was applied to TBI patients and Mx to patients with non-traumatic diseases, the overall strongest correlations to outcome were observed.

## Conclusion

Outcome predictive values of PRx and Mx depend on patient characteristics. Further studies with larger populations should be performed on this subject to allow recommendations for an index-specific clinical use.

## References

[1] Enevoldsen EM, Jensen FT (1978). Autoregulation and CO_2_ responses of cerebral blood flow in patients with severe head injury. J Neurosurg.

[2] Lassen NA (1974). Control of cerebral circulation in health and disease. Circ Res.

[3] Aaslid R, Lindegaard KF, Sorteberg W, Nornes H (1989). Cerebral autoregulation dynamics in humans. Stroke.

[4] Czosnyka M, Smielewski P, Kirkpatrick P, Menon DK, Pickard JD (1996). Monitoring of cerebral autoregulation in head-injured patients. Stroke.

[5] Czosnyka M, Smielewski P, Kirkpatrick P, Laing RJ, Menon D, Pickard JD (1997). Continuous assessment of the cerebral vasomotor reactivity in head injury. Neurosurgery.

[6] Newell DW, Aaslid R, Lam A, Mayberg TS, Winn HR (1994). Comparison of flow and velocity during dynamic autoregulation testing in humans. Stroke.

[7] Brady KM, Easley RB, Kibler K, Kaczka DW, Andropoulos D, Fraser CD, Smielewski P, Czosnyka M, Adams GJ, Rhee CJ, Rusin CG (2012). Positive end-expiratory pressure oscillation facilitates brain vascular reactivity monitoring. J Appl Physiol.

[8] Budohoski KP, Reinhard M, Aries MJ, Czosnyka Z, Smielewski P, Pickard JD, Kirkpatrick PJ, Czosnyka M (2012). Monitoring cerebral autoregulation after head injury. Which component of transcranial Doppler flow velocity is optimal?. Neurocrit Care.

[9] Sánchez-Porras R, Santos E, Czosnyka M, Zheng Z, Unterberg AW, Sakowitz OW (2012). ‘Long’ pressure reactivity index (L-PRx) as a measure of autoregulation correlates with outcome in traumatic brain injury patients. Acta Neurochir (Wien).

[10] Sorrentino E, Budohoski KP, Kasprowicz M, Smielewski P, Matta B, Pickard JD, Czosnyka M (2011). Critical thresholds for transcranial Doppler indices of cerebral autoregulation in traumatic brain injury. Neurocrit Care.

[11] Sorrentino E, Diedler J, Kasprowicz M, Budohoski KP, Haubrich C, Smielewski P, Outtrim JG, Manktelow A, Hutchinson PJ, Pickard JD, Menon DK, Czosnyka M (2012). Critical thresholds for cerebrovascular reactivity after traumatic brain injury. Neurocrit Care.

[12] Diedler J, Sykora M, Rupp A, Poli S, Karpel-Massler G, Sakowitz O, Steiner T (2009). Impaired cerebral vasomotor activity in spontaneous intracerebral hemorrhage. Stroke.

[13] Reinhard M, Neunhoeffer F, Gerds TA, Niesen WD, Buttler KJ, Timmer J, Schmidt B, Czosnyka M, Weiller C, Hetzel A (2010). Secondary decline of cerebral autoregulation is associated with worse outcome after intracerebral hemorrhage. Intensive Care Med.

[14] Barth M, Woitzik J, Weiss C, Muench E, Diepers M, Schmiedek P, Kasuya H, Vajkoczy P (2010). Correlation of clinical outcome with pressure-, oxygen-, and flow-related indices of cerebrovascular reactivity in patients following aneurysmal SAH. Neurocrit Care.

[15] Aries MJ, Czosnyka M, Budohoski KP, Steiner LA, Lavinio A, Kolias AG, Hutchinson PJ, Brady KM, Menon DK, Pickard JD, Smielewski P (2012). Continuous determination of optimal cerebral perfusion pressure in traumatic brain injury. Crit Care Med.

[16] Diedler J, Santos E, Poli S, Sykora M (2014). Optimal cerebral perfusion pressure in patients with intracerebral hemorrhage: an observational case series. Crit Care.

[17] Lazaridis C, Smielewski P, Steiner LA, Brady KM, Hutchinson P, Pickard JD, Czosnyka M (2013). Optimal cerebral perfusion pressure: are we ready for it?. Neurol Res.

[18] Steiner LA, Czosnyka M, Piechnik SK, Smielewski P, Chatfield D, Menon DK, Pickard JD (2002). Continuous monitoring of cerebrovascular pressure reactivity allows determination of optimal cerebral perfusion pressure in patients with traumatic brain injury. Crit Care Med.

[19] Schmidt B, Schwarze JJ, Weinhold M, Lezaic V, Czosnyka M, Klingelhöfer J (2014). Impaired autoregulation is associated with mortality in severe cerebral diseases. IJCNMH.

[20] Schmidt B, Czosnyka M, Raabe A, Yahya H, Schwarze JJ, Sackerer D, Sander D, Klingelhöfer J (2003). Adaptive noninvasive assessment of intracranial pressure and cerebral autoregulation. Stroke.

[21] Mudholkar GS, McDermott M, Srivastava DK (1992). A test of p-variate normality. Biometrika.

[22] DeLong ER, DeLong DM, Clarke-Pearson DL (1988). Comparing the areas under two or more correlated receiver operating characteristic curves: a nonparametric approach. Biometrics.

[23] Wang EC, Ang BT, Wong J, Lim J, Ng I (2006). Characterization of cerebrovascular reactivity after craniectomy for acute brain injury. Br J Neurosurg.

[24] Timofeev I, Czosnyka M, Nortje J, Smielewski P, Kirkpatrick P, Gupta A, Hutchinson P (2008). Effect of decompressive craniectomy on intracranial pressure and cerebrospinal compensation following traumatic brain injury. J Neurosurg.

[25] Maset AL, Marmarou A, Ward JD, Choi S, Lutz HA, Brooks D, Moulton RJ, DeSalles A, Muizelaar JP, Turner H, Young HF (1987). Pressure-volume index in head injury. J Neurosurg.

[26] Gray WJ, Rosner MJ (1987). Pressure-volume index as a function of cerebral perfusion pressure. Part 1: the effects of cerebral perfusion pressure changes and anesthesia. J Neurosurg.

[27] Gray WJ, Rosner MJ (1987). Pressure-volume index as a function of cerebral perfusion pressure. Part 2: the effects of low cerebral perfusion pressure and autoregulation. J Neurosurg.

[28] Budohoski KP, Czosnyka M, de Riva N, Smielewski P, Pickard JD, Menon DK, Kirkpatrick PJ, Lavinio A (2012). The relationship between cerebral blood flow autoregulation and cerebrovascular pressure reactivity after traumatic brain injury. Neurosurgery.

[29] Vittinghoff E, McCulloch CE (2006). Relaxing the rule of ten events per variable in logistic and Cox regression. Am J Epidemiol.

